# Aortic Arch Tortuosity Index Is Associated with Aortic Enlargement After Thoracic Endovascular Aortic Repair for Left Subclavian Artery Reconstruction Using a Single-Branched Stent-Graft in Type B Aortic Dissection: A Multicenter Retrospective Study

**DOI:** 10.3390/jcm15114139

**Published:** 2026-05-27

**Authors:** Yapeng Zhu, Long Cao, Wei Guo, Hongpeng Zhang

**Affiliations:** 1 Department of Vascular Surgery, The First Medical Center, Chinese PLA General Hospital, Beijing 100853, China; zhuyapeng09@163.com (Y.Z.); drcaolong@163.com (L.C.); guoweiplagh@sina.com (W.G.); 2 Medical School, Chinese PLA, Beijing 100853, China; 3 Department of General Surgery, The 983rd Hospital of Joint Logistic Support Force of PLA, Tianjin 300142, China

**Keywords:** type B aortic dissection, thoracic endovascular aortic repair, aortic enlargement, tortuosity index, aortic arch

## Abstract

**Objective:** To investigate the relationship between the aortic arch Tortuosity Index (TI) and post-TEVAR aortic enlargement in patients with type B aortic dissection (TBAD) requiring left subclavian artery (LSA) reconstruction with a single-branched stent-graft. **Methods:** We retrospectively analyzed 120 patients enrolled in a prospective multicenter clinical trial between December 2020 and November 2021. The aortic arch TI was measured to quantify arch tortuosity. The study evaluated the independent association between TI and the risk of post-TEVAR aortic enlargement using multivariable Cox regression analysis. Furthermore, we estimated enlargement-free survival in patients stratified by TI levels and verified the diagnostic performance of TI for both thoracic and abdominal aortic enlargement. **Results:** The mean age was 57.0 ± 10.6 years. Multivariable Cox regression revealed TI was independently associated with both thoracic (HR, 3.8; 95% CI, 2.2–6.6; *p* < 0.001) and abdominal aortic enlargement (HR, 2.5; 95% CI, 1.6–4.1; *p* < 0.001). Kaplan–Meier survival curves demonstrated that freedom from aortic enlargement was significantly lower in the High-TI group (*p* < 0.001). ROC curves confirmed excellent discriminative ability for thoracic (AUC: 0.857, 95% CI, 0.775–0.940) and abdominal (AUC: 0.833, 95% CI, 0.756–0.910) enlargement. **Conclusions:** In TBAD patients requiring LSA reconstruction with a single-branched stent-graft, the aortic arch tortuosity is independently associated with post-TEVAR aortic enlargement. Preoperative measurement of the aortic arch tortuosity contributes to risk stratification in patients with TBAD and may identify those requiring intensified postoperative surveillance.

## 1. Introduction

The treatment paradigm for type B aortic dissection (TBAD) has been revolutionized by thoracic endovascular aortic repair (TEVAR) [1]. Compared to traditional open surgery, it significantly reduces perioperative mortality and morbidity [2]. Despite early benefits, TEVAR has a relatively high rate of reintervention, with post-TEVAR aortic enlargement being a major contributing factor [3]. Aortic enlargement may progress to aneurysm formation, posing a significant risk of fatal rupture. Earlier research has demonstrated that post-TEVAR aortic enlargement is associated with distal stent-graft-induced new entry (SINE), retrograde type A dissection (RTAD), false lumen-perfused intercostal arteries, distal tears, and false lumen thrombosis status [4,5,6,7,8]. However, these studies primarily focus on local anatomical features or distal anchorage site characteristics, to some extent neglecting the influence of overall aortic geometry—particularly aortic arch.

Numerous metrics describe aortic arch geometry, including radius of curvature, angulation, and tortuosity index (TI) [9,10]. Among various arch geometry metrics, the TI is a classic, widely accepted metric for quantifying aortic tortuosity, typically defined as the ratio of the vessel’s centerline length to the straight-line distance between its two endpoints. Higher TI values indicate more severe aortic arch tortuosity. Our previous studies have confirmed that patients with TBAD exhibit significantly higher aortic arch TI than controls [11], and TI is considered as an independent anatomical risk factor for TBAD development [9]. This raises a critical clinical question: given that high aortic arch tortuosity is one of the anatomical foundations for TBAD occurrence, does this inherent unfavorable morphology similarly impact patient prognosis post-TEVAR?

However, a systematic evaluation of the correlation between aortic arch TI and post-TEVAR aortic enlargement in patients with TBAD remains lacking. Consequently, the primary objective of this research is to clarify the association between the aortic arch TI and the incidence of aortic enlargement post-TEVAR.

## 2. Materials and Methods

### 2.1. Study Population

This was a retrospective analysis of data from a prospective, multicenter clinical trial involving 120 patients who underwent LSA reconstruction using single-branched stent-graft between December 2020 and November 2021. The detailed protocol and primary results of this trial have been previously published [12]. The study cohort comprised patients who met the following criteria: (1) age ranging from 18 to 80 years; (2) TBAD requiring LSA revascularization; (3) proximal landing zone diameter 18–42 mm and length ≥ 15 mm; LSA diameter 6–20 mm; and femoral access diameter ≥6.6 mm; and (4) distance from left common carotid artery to LSA ≥ 5 mm and from LSA to left vertebral artery ≥ 15 mm. Criteria for exclusion included: (1) hereditary connective tissue disorders (such as Marfan or Ehlers–Danlos syndrome); (2) prohibitive vascular access anatomy; and (3) hypersensitivity to nitinol or iodine contrast. The Institutional Review Board waived informed consent for patients undergoing retrospective data review.

### 2.2. Image Processing

Image analysis was performed using 3mensio Vascular software (v8.1; Medical Imaging, Amsterdam, The Netherlands) on all computed tomography angiography (CTA) datasets stored in DICOM format. Following aortic segmentation, the true lumen centerline (CLL) was automatically generated by placing three seed points (at the aortic root, aortic arch, and abdominal bifurcation). This automatically generated CLL was subsequently reviewed and manually adjusted as necessary. The finalized centerline was then used to generate curved multiplanar reformat images, extending from the aortic valve annulus to the aortic bifurcation, for all subsequent geometric measurements.

### 2.3. Anatomical Landmarks

**Figure 1:**
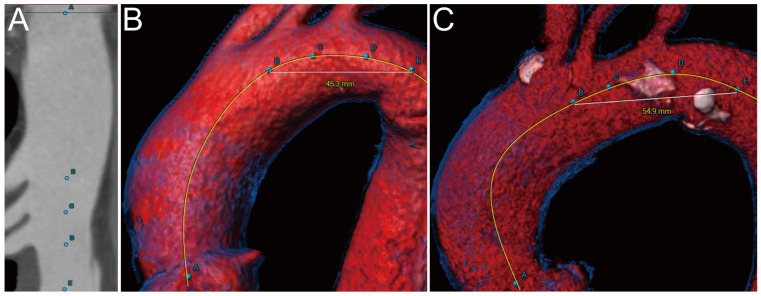
Definition of anatomical landmarks and illustration of aortic arch tortuosity using 3mensio Vascular software. (**A**) Stretched view (curved multiplanar reformat) perpendicular to the CLL, showing the five standardized anatomical landmarks (A–E). Point A: sinotubular junction; Point B: distal margin of the innominate artery; Point C: distal margin of the left common carotid artery; Point D: distal margin of the LSA; Point E: 2 cm distal to the LSA. (**B**) A representative reconstruction with a high TI, exhibiting a steep and highly tortuous aortic arch. (**C**) A representative reconstruction with a low TI, showing a flatter tortuous aortic arch. LSA = left subclavian arteries; left subclavian arteries.

Five standardized anatomical landmarks (A–E) were identified on the stretched view (curved multiplanar reformat) perpendicular to the CLL (Figure 1A). These landmarks were defined as follows: Point A denoted the sinotubular junction, while Points B, C, and D marked the distal margins of the innominate, left common carotid, and LSA, respectively. Point E was set 2 cm distal to the LSA. Based on these markers, the aortic arch was delineated from B to E. Correspondingly, Ishimaru Zones were classified as: Zone 0 (A–B), Zone 1 (B–C), Zone 2 (C–D), and Zone 3 (D–E).

### 2.4. Anatomical Parameters

Baseline dissection characteristics were assessed, including the diameter of the primary intimal tear, total dissection length, the number of distal intimal tears and the total number of intimal tears. Aortic arch geometry was classified as Type I, II, or III based on the vertical distance between the origin of the innominate artery and the top of the aortic arch [13]. To quantify the false lumen thrombosis status, a total patency score was calculated for Zones 3–9. Based on preoperative CTA, each zone was assigned a score according to its patency: patent (3 points), partial thrombosis (2 points), or complete thrombosis (1 point). Zones not involved by the false lumen (FL) were assigned 0 points. The final score was derived by summing these segmental scores. Additionally, the preoperative maximum diameters of the descending thoracic aorta (DTA) and abdominal aorta (AA), along with the corresponding true lumen (TL) and FL diameters at these measurement planes, were measured. Patients were stratified based on the interval from symptom onset to TEVAR; those treated within 14 days were classified as the acute phase, while those treated after 14 days were categorized as the chronic phase.

### 2.5. Tortuosity Index

To quantify the overall tortuosity of the aortic arch, the TI from Point B to Point E was calculated. This index was defined as the ratio of the CLL length to the straight-line distance between these two endpoints [9]. The straight-line distance was calculated via the software’s built-in “distance between 2 marked points” algorithm [14]. Representative reconstructions generated using 3mensio Vascular software illustrating an aortic arch with high tortuosity (steep arch) and one with low tortuosity (flat arch) are shown in Figure 1B and Figure 1C, respectively.

### 2.6. End Points

Postoperative surveillance included CTA at 1, 6, and 12 months and annually thereafter. The primary outcome of this study is aortic enlargement, encompassing thoracic aortic enlargement (TAE) and abdominal aortic enlargement (AAE), defined as an increase in the overall maximum aortic diameter ≥5 mm compared to preoperative measurements [7,15].

### 2.7. Measurement Reproducibility

Measurements were separate by 2 observers for 40 CTAs randomly selected from the cohort to evaluate the interobserver repeatability using the intra-class correlation coefficients and Bland–Altman plots (full details appear in Table S1 and Figure S1). During the entire image analysis process, the two independent operations were completely blinded to the patients’ clinical outcomes.

### 2.8. Statistical Analysis

Normally and non-normally distributed continuous variables are presented as the mean ± standard deviation (SD) and median (interquartile range [IQR]), respectively. Categorical variables are presented as number and percentage. To evaluate the independent association between the preoperative aortic arch TI and post-TEVAR aortic enlargement, we constructed multivariable Cox proportional hazards regression models. TI was entered into the regression models as a continuous variable (scaled by 100) and categorical variable (Low-TI and High-TI groups based on the cut-off value). The optimal cut-off value for the aortic arch TI was determined as 101.2 based on the method that yields the minimum *p*-value for group comparison, utilizing the cut-off package https://github.com/yikeshu0611/cutoff (accessed on 20 March 2026) in R Statistical Software. Data are expressed as hazard ratios (HR) with 95% confidence intervals (CI). The cumulative rates of freedom from TAE and AAE were visualized using Kaplan–Meier (KM) survival curves. To test for non-linearity, restricted cubic spline (RCS) models were fitted using knots positioned at the 10th, 50th, and 90th percentiles. All multivariable models were adjusted for potential confounders selected based on clinical relevance and the previous literature, including age, sex, total patency score, and number of distal tears [9,16]. Furthermore, subgroup analyses stratified by dissection phase and preoperative maximum aortic diameter were conducted to verify the consistency of the results. We performed receiver operating characteristic (ROC) curve analysis to evaluate the diagnostic performance of the aortic arch TI for 1-year binary enlargement, calculating the area under the curve (AUC). To handle missing values, multiple imputation was applied for variables with less than 20% missing data to ensure robustness (Table S2). All statistical analyses were performed using R Statistical Software (Version 4.2.2; The R Foundation for Statistical Computing, Vienna, Austria) and the FreeStatistics Analysis Platform (Version 2.3, Beijing, China; http://www.clinicalscientists.cn/freestatistics, accessed on 20 March 2026). A two-sided *p*-value of less than 0.05 was considered statistically significant.

## 3. Results

### 3.1. Baseline Demographic and Preoperative Anatomical Characteristics

Table 1 summarizes the baseline characteristics of the 120 patients. The mean age was 57.0 ± 10.6 years, and 101 patients (84.2%) were male. Hypertension was the most frequent comorbidity (92.5%), followed by smoking (40.8%). No statistically significant differences were observed between the Low-TI and High-TI groups regarding age, sex, body mass index, or comorbidities (all *p* > 0.05).

The preoperative anatomical characteristics measured on CTA are presented in Table 2. The cohort comprised 107 patients (89.2%) in the acute phase and 13 patients (10.8%) in the chronic phase. The mean maximum diameters of the DTA and AA were 40.4 ± 6.8 mm and 29.0 ± 4.9 mm, respectively. The mean scaled TI was significantly higher in the High-TI group (101.9 ± 0.6) compared to the Low-TI group (100.5 ± 0.6) (*p* < 0.001). Furthermore, no statistically significant differences were observed between the two groups regarding other anatomical parameters.

### 3.2. One-Year Clinical Outcomes and Postoperative Anatomical Data

Table 3 details the clinical outcomes and anatomical measurements at 1-year follow-up. The mean follow-up duration was 11.0 ± 2.8 months. The overall all-cause mortality rate was 4.2% (*n* = 5), with no significant difference between the Low-TI and High-TI groups (*p* = 0.672). Similarly, the incidence of postoperative complications, including endoleak, RTAD, SINE, and neurologic events, was low and comparable between the two groups (all *p* > 0.05). The High-TI group exhibited a significantly higher rate of TAE and AAE compared with the Low-TI group (*p* < 0.001). Moreover, the High-TI group had significantly larger postoperative DTA overall diameters (38.6 ± 6.8 vs. 35.1 ± 5.9 mm, *p* = 0.004) and TL diameters (30.7 ± 5.6 vs. 28.5 ± 4.3 mm, *p* = 0.019). AAE occurred more frequently in the High-TI group (45.2 vs. 3.4%, *p* < 0.001). This was characterized by a significantly larger abdominal FL diameter in the High-TI group median 14.5 mm (IQR 3.8, 20.1) compared with the Low-TI group of 9.2 mm (IQR 1.5, 15.2) (*p* = 0.020), despite similar overall and TL diameters (*p* > 0.05).

### 3.3. The Association Between Aortic Arch TI and Aortic Enlargement

#### 3.3.1. Multivariable Cox Regression Analysis

After adjustment for age, sex, total patency score, and distal intimal tear numbers, multivariable Cox regression analysis demonstrated TI remained independently associated with TAE and AAE (Table 4). When analyzed as a continuous variable, TI was associated with TAE (HR, 3.8; 95% CI, 2.2–6.6; *p* < 0.001) and AAE (HR, 2.5; 95% CI,1.6–4.1; *p* < 0.001). In categorical analysis, high TI was identified as an independent risk factor for TAE (HR, 4.5; 95% CI, 1.5–13.6; *p* = 0.010) and AAE (HR, 10.8; 95% CI, 2.6–45.5; *p* < 0.001).

#### 3.3.2. Kaplan–Meier Survival Curves

**Figure 2:**
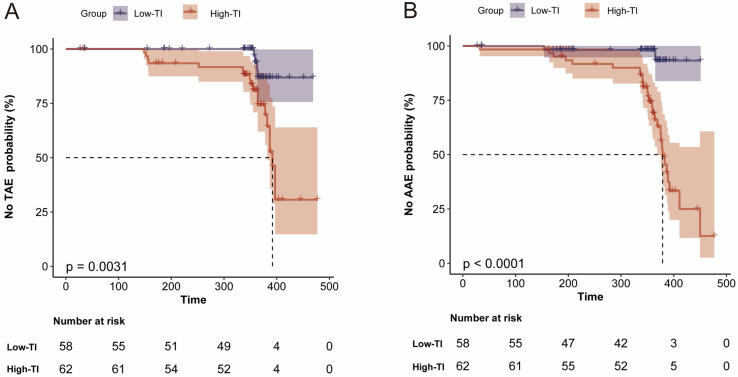
Aortic enlargement according to aortic arch TI. (**A**) Thoracic aortic enlargement, (**B**) abdominal aortic enlargement. TI = tortuosity index.

KM survival analysis showed that freedom from TAE and AAE was significantly lower in the High-TI group compared to the Low-TI group (TAE: Log-rank *p* = 0.003, Figure 2A; AAE: Log-rank *p* < 0.001, Figure 2B).

#### 3.3.3. Restricted Cubic Spline

RCS analysis revealed a linear relationship between aortic arch TI and the risk of TAE and AAE (*p* for non-linearity > 0.05; Figure S2A,B).

#### 3.3.4. Subgroup Analysis

**Figure 3:**
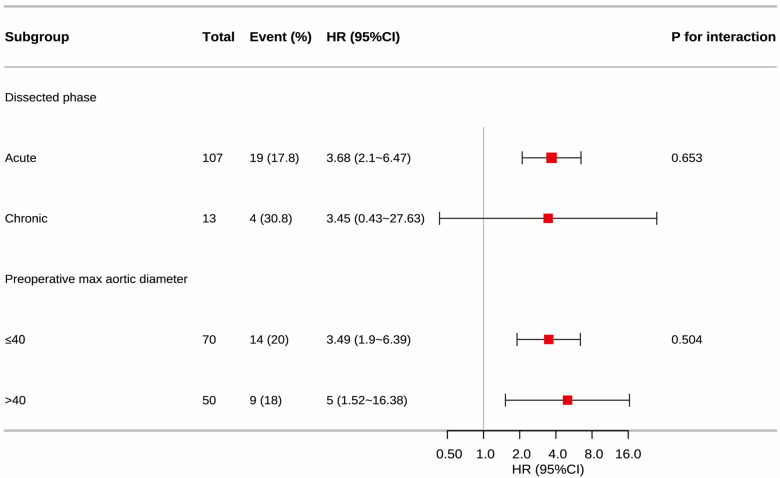
Subgroup analysis of aortic enlargement according to aortic arch TI, stratified by dissection phase and preoperative aortic diameter. HR = hazard ratio; CI = confidence interval; TI = tortuosity index.

No significant interaction was observed for dissection phase (*p* for interaction = 0.653) and aortic diameter (*p* for interaction = 0.504). High TI associated with aortic enlargement in both the acute (HR, 3.68) and chronic phases (HR, 3.45). Furthermore, the association persisted in patients with small aortic diameters (≤40 mm, HR, 3.49) as well as those with larger diameters (>40 mm; HR, 5.00) (Figure 3).

### 3.4. Receiver Operating Characteristic Curve Analysis

**Figure 4:**
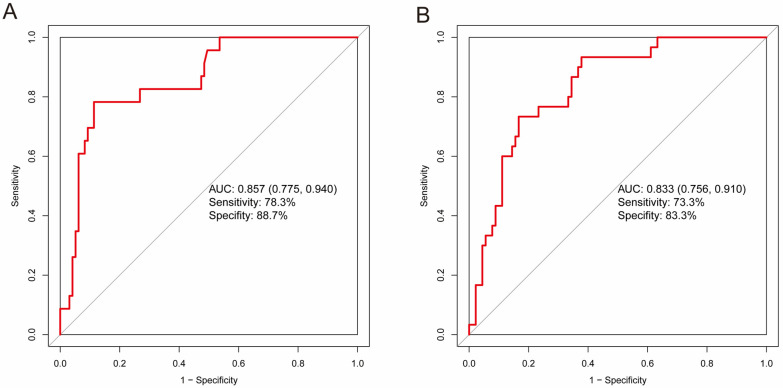
Receiver operating characteristic curves of aortic arch TI for predicting aortic enlargement. (**A**) Thoracic aortic enlargement, (**B**) abdominal aortic enlargement. TI = tortuosity index; AUC = area under the curve.

ROC analysis confirmed robust diagnostic performance of TI for both TAE (AUC: 0.857; sensitivity: 78.3%; specificity: 88.7%) and AAE (AUC: 0.833; sensitivity: 73.3%; specificity: 83.3%) (Figure 4).

## 4. Discussion

Aortic enlargement represents an early pathological manifestation of aneurysmal degeneration [17]. Post-TEVAR aortic enlargement is not merely a morphological finding but a critical indicator of poor prognosis. Resch TA et al. reported that patients with TBAD who exhibit aortic enlargement have a risk of 13.4% to 19.4% of developing aneurysm formation and fatal rupture [18]. This underscores the importance of early prevention.

The aortic arch, connecting the ascending and descending aorta, features complex anatomy that remains a focus of vascular research. It is widely accepted that precise preoperative assessment of aortic arch tortuosity reduces procedural difficulty and improves prognosis [16]. The TI is a key metric for quantifying global arch tortuosity. Piazza et al. [19] identified TI as a predictor of complications following branched endovascular repair of thoracoabdominal aortic aneurysms (TAAA). Similarly, Sugimoto et al. [20] reported TI as a risk factor for renal artery stenosis and occlusion in TAAA. In this study, we systematically confirmed that aortic arch TI is an independent risk factor for AAE and TAE post-TEVAR in this cohort treated with a Zone 2 single-branched stent-graft.

Endovascular repair involving the aortic arch remains technically challenging in patients with TBAD. Generally, increased arch curvature predicts inadequate stent graft apposition, radiologically manifesting as a “bird-beak” configuration [21]. This suboptimal conformation subsequently predisposes patients to type I endoleaks and aortic enlargement. While both type I and type II endoleaks are known to cause enlargement, our study revealed a significantly higher incidence of TAE in the High-TI group (30.6 vs. 6.9%), despite there being no statistical difference in the rate of detectable endoleaks. This suggests that, in highly tortuous anatomies, post-TEVAR aortic enlargement may involve mechanisms beyond overt endoleaks. The risk of TAE post-TEVAR rises with increasing aortic arch TI, but the mechanism remains unclear. The following factors may contribute: First, the deployment of rigid stent grafts into a curved arch generates a spring-back force [22,23], exerting continuous stress on the aortic wall. A high TI implies aortic elongation [24], which imposes traction forces on the endograft, a phenomenon De Masi et al. [24] described as lateral traction. Our data indicate that, despite comparable preoperative TL diameters (19.3 ± 6.7 vs. 19.8 ± 5.8 mm, *p* = 0.650), the TL diameter in the High-TI group was significantly larger than that in the Low-TI group at the 1-year postoperative follow-up (30.7 ± 5.6 vs. 28.5 ± 4.3 mm, *p* = 0.019). This discrepancy suggests that, in the High-TI group, the stent graft not only restored TL patency but also could amplify the spring-back force of the rigid device as the arch curvature increased, leading to over-distension of the TL. This over-distension may contribute to a significantly larger postoperative total DTA diameter in the High-TI group (38.6 ± 6.8 mm vs. 35.1 ± 5.9 mm, *p* = 0.004). While TL expansion is a therapeutic goal, the excessive radial force in High-TI anatomies may contribute to total vessel enlargement, shifting the outcome from favorable remodeling to expansive stress. Consequently, this could impede the physiological shrinkage of the aortic wall and, in some patients, could potentially lead to TAE. Second, as illustrated by Li et al. [25] in their computational model of thoracic aortic pathology, maintaining aortic integrity relies on intimal cells actively sensing and adapting to mechanical stress. In High-TI anatomies, the deployment of rigid stents can impose persistent, non-physiological radial stress on the aortic wall, leading to severe compliance mismatch. This chronic mechanical disturbance may disrupt the mechanosensing set-point of aortic wall cells, potentially triggering adverse remodeling pathways. Furthermore, driven by cardiac and respiratory movements, the persistent dynamic interaction between the stent and the vessel wall induces chronic local inflammation [26]. This process has been associated with extracellular matrix degradation and wall weakening [27]. Finally, the presence of occult endoleaks cannot be excluded in tortuous aortic arches [28]; micro-channels undetectable by standard CTA may persist.

Our findings on the impact of arch geometry are strongly supported by the work of Cho et al. [15], who identified Zone 2 landing as an independent predictor of aortic enlargement. However, their study did not explain the geometric mechanism, largely because they excluded patients with steep aortic arch from their cohort. Our study offers a potential mechanistic explanation. The “Zone 2 risk” observed by Cho et al. closely related to high aortic arch tortuosity, as Zone 2 anatomically represents the segment of maximal curvature. Therefore, the adverse outcomes could be largely driven by the mechanical incompatibility rather than the landing zone itself. Furthermore, in standard TEVAR anchored in Zone 3 or 4, the stent spring-back and compliance mismatch are theoretically attenuated. Consequently, the risk of high-TI-related adverse remodeling may be lower compared to Zone 2 landings.

Based on our clinical experience and knowledge, the incidence of AAE post-TEVAR is higher than TAE, and distal tears are closely associated with AAE. The results of our study show that distal tears are present in both the high-TI and low-TI groups. However, an interesting observation is that, at the one-year follow-up, FL expansion was more apparent in the High-TI group. Several plausible explanations exist: First, patients with TBAD often exhibit anatomical vulnerabilities in the AA, including patent false lumens, fragile intimal flaps, and distal tears. These anatomical weaknesses render the AA particularly susceptible to mechanical stress and hemodynamic impact. Concurrently, previous computational fluid dynamics (CFD) studies have demonstrated that increased tortuosity of the aortic arch may induce abnormally high-energy helical flow, significantly elevating wall shear stress (WSS) [29]. Post-TEVAR, this high-kinetic-energy abnormal flow ejecting from the graft tip could directly impact the AA. This process has been shown to alter endothelial cell transcription profiles, potentially aggravating existing pathology, and promotes AAE [30]. Second, Nomura et al. [22] and Li et al. [31] emphasized that graft traction damages the distal vascular intima, inducing SINE. While neither group in this study showed a significant increase in SINE, this mechanical stress could potentially act on the distal vessel wall in a more invisible manner, contributing to increased pressure within the FL, as similarly noted by Dong et al. [32]. Furthermore, Andacheh et al. [33] reported dilation of subrenal aortic FL diameter and total abdominal aortic volume post-TEVAR. Combined with our findings, this suggests the need for heightened caution regarding abdominal FL and timely implementation of appropriate interventions.

Aortic diameter and dissection staging are factors influencing aortic remodeling. Our analysis revealed that the association between TI and both TAE and AAE trended in the same direction across all subgroups. This suggests that the tortuosity of the aortic arch may be a more significant factor influencing aortic enlargement than diameter and time. Furthermore, ROC curve analysis further validated the value of TI, demonstrating excellent sensitivity and specificity in identifying patients with aortic enlargement, suggesting its significant potential as a routine preoperative assessment tool.

Aortic enlargement is a chronic condition that may progress to aneurysm formation, posing a risk of rupture. Therefore, early preventive interventions are necessary. Currently, following proximal TEVAR procedures, endovascular methods may be required to promote favorable aortic remodeling. Several advanced solutions have been proposed, ranging from novel device designs like composite stents to specific techniques such as PETTICOAT and STABILIZE. These approaches may be particularly beneficial for patients with high aortic arch tortuosity.

## 5. Limitations

Our study has several limitations. First, the retrospective design and one-year follow-up may be insufficient to capture the true long-term incidence of aortic enlargement. Second, evaluating arch morphology solely via TI warrants future incorporation of multi-dimensional metrics like angulation. Third, because all patients received Zone 2 single-branch grafts, generalizability to other zones or populations needs validation. Fourth, the limited outcome events (23 TAE and 30 AAE) restricted covariate adjustment in multivariable models. Finally, the precise biomechanical and hemodynamic impacts of arch tortuosity on stent graft performance were not assessed. Future finite element analysis (FEA) and CFD studies are needed to explicitly quantify these complex interactions. Despite these constraints, our findings clearly demonstrate that aortic arch TI is a critical determinant of post-TEVAR aortic enlargement in patients with TBAD.

## 6. Conclusions

Increased aortic arch tortuosity is independently associated with post-TEVAR aortic enlargement in this cohort. Incorporating aortic arch tortuosity into preoperative assessment may be valuable for risk stratification and optimizing procedural planning to improve patient prognosis. Future CFD and FEA studies are warranted to elucidate the underlying hemodynamic mechanisms of the aortic arch.

## Figures and Tables

**Table 1:** Characteristics of preoperative patients.

Variable	Overall (*n* = 120)	Low-TI (*n* = 58)	High-TI (*n* = 62)	*p*-Value
Age, (years) ^a^	57.0 ± 10.6	56.9 ± 11.2	57.0 ± 10.2	0.969
BMI, (kg/m^2^) ^a^	26.1 ± 3.4	26.0 ± 3.7	26.2 ± 3.1	0.769
Male ^b^	101 (84.2)	51 (87.9)	50 (80.6)	0.275
Smoking ^b^	49 (40.8)	25 (43.1)	24 (38.7)	0.625
Hypertension ^b^	111 (92.5)	52 (89.7)	59 (95.2)	0.312
Diabetes ^b^	6 (5.0)	1 (1.7)	5 (8.1)	0.208
Hyperlipemia ^b^	4 (3.3)	2 (3.4)	2 (3.2)	1
Coronary heart disease ^b^	6 (5.0)	4 (6.9)	2 (3.2)	0.428

Abbreviations: BMI: body mass index; TI: tortuosity index. ^a^ Continuous data are presented as mean ± standard deviation. ^b^ Categorical variables are presented as number and percentage (%).

**Table 2:** Preoperative anatomical data.

Variable	Overall (*n* = 120)	Low-TI (*n* = 58)	High-TI (*n* = 62)	*p*-Value
Dissected length (mm) ^a^	391.9 ± 111.2	403.9 ± 114.8	380.7 ± 107.4	0.255
Primary intimal tear diameter (mm) ^a^	9.3 ± 4.3	9.0 ± 4.3	9.5 ± 4.3	0.517
DTA max overall diameter (mm) ^a^	40.4 ± 6.8	40.3 ± 5.4	40.5 ± 8.0	0.915
DTA max TL diameter (mm) ^a^	19.6 ± 6.3	19.3 ± 6.7	19.8 ± 5.8	0.650
DTA max FL diameter (mm) ^a^	20.8 ± 8.4	20.6 ± 8.3	21.0 ± 8.7	0.800
AA max overall diameter (mm) ^a^	29.0 ± 4.9	29.0 ± 4.7	29.0 ± 5.1	0.939
AA max TL diameter (mm) ^a^	15.4 ± 5.7	14.9 ± 5.3	15.9 ± 6.2	0.337
AA max FL diameter (mm) ^a^	13.6 ± 7.5	13.1 ± 7.3	14.2 ± 7.8	0.436
Total thrombosis score (Zones 3–9) ^a^	16.1 ± 5.7	16.9 ± 5.4	15.4 ± 6.0	0.142
Total intimal tear number (Zones 3–9) ^c^	2.0 (1.0, 3.0)	2.0 (1.0, 3.0)	2.0 (1.0, 2.8)	0.482
Distal intimal tear number (Zones 5–9) ^c^	1.0 (0.0, 1.2)	1.0 (0.0, 2.0)	1.0 (0.0, 1.0)	0.446
TI ^a^	101.2 ± 0.9	100.5 ± 0.6	101.9 ± 0.6	<0.001
AD phase (Acute) ^b^	107 (89.2)	52 (89.7)	55 (88.7)	0.868
Arch type ^b^				0.446
Type I	19 (15.8)	10 (17.2)	9 (14.5)	
Type II	75 (62.5)	33 (56.9)	42 (67.7)	
Type III	26 (21.7)	15 (25.9)	11 (17.7)	

Abbreviations: TI, tortuosity index; AD, aortic dissection; DTA, descending thoracic aorta; AA, abdominal aorta; TL, true lumen; FL, false lumen. ^a^ Data are presented as mean ± standard deviation. ^b^ Categorical variables are presented as number and percentage (%). ^c^ Data with skewed distribution are presented as median (IQR).

**Table 3:** Postoperative clinical outcomes and anatomical data.

Variable	Overall (*n* = 120)	Low-TI (*n* = 58)	High-TI (*n* = 62)	*p*-Value
DTA max overall diameter (mm) ^a^	36.9 ± 6.6	35.1 ± 5.9	38.6 ± 6.8	0.004
DTA max TL diameter (mm) ^a^	29.6 ± 5.1	28.5 ± 4.3	30.7 ± 5.6	0.019
DTA max FL diameter (mm) ^c^	4.3 (0.0, 12.0)	4.0 (0.0, 11.5)	4.6 (0.0, 12.8)	0.654
AA max overall diameter (mm) ^a^	29.9 ± 5.8	29.5 ± 5.2	30.4 ± 6.3	0.412
AA max TL diameter (mm) ^a^	18.8 ± 4.5	19.2 ± 4.2	18.4 ± 4.8	0.325
AA max FL diameter (mm) ^c^	11.4 (2.3, 17.7)	9.2 (1.5, 15.2)	14.5 (3.8, 20.1)	0.020
Death ^b^	5 (4.2)	3 (5.2)	2 (3.2)	0.672
Complications				
Endoleak ^b^	5 (4.2)	2 (3.4)	3 (4.8)	1
RTAD ^b^	3 (2.5)	1 (1.7)	2 (3.2)	1
SINE ^b^	1 (0.8)	1 (1.7)	0 (0)	0.483
Stroke ^b^	3 (2.5)	2 (3.4)	1 (1.6)	0.609
Respiratory failure ^b^	2 (1.7)	2 (3.4)	0 (0)	0.232
Spinal cord ischemia ^b^	1 (0.8)	0 (0)	1 (1.6)	1
Stenosis or occlusion ^b^	1 (0.8)	0 (0)	1 (1.6)	1
Aortic intimal intussusception ^b^	1 (0.8)	0 (0)	1 (1.6)	1
Aortic enlargement ^b^				
TAE	23 (19.2)	4 (6.9)	19 (30.6)	<0.001
AAE	30 (25.0)	2 (3.4)	28 (45.2)	<0.001

Abbreviations: RTAD, retrograde type A aortic dissection; SINE, stent-induced new entry; TAE, thoracic aortic enlargement; AAE, abdominal aortic enlargement; DTA, thoracic descending aorta; AA, abdominal aorta; TL, true lumen; FL, false lumen; TI, tortuosity index. ^a^ Data are presented as mean ± standard deviation. ^b^ Categorical variables are presented as number and percentage (%). ^c^ Data with skewed distribution are presented as median (IQR).

**Table 4:** Multivariable Cox regression analysis for aortic enlargement at 1-year follow-up.

TI	TAE (23 Events)				AAE (30 Events)			
	Model 1		Model 2		Model 1		Model 2	
	HR (95% CI)	*p*-Value	HR (95% CI)	*p*-Value	HR (95% CI)	*p*-Value	HR (95% CI)	*p*-Value
Continuous	3.7 (2.2~6.2)	<0.001	3.8 (2.2~6.6)	<0.001	2.6 (1.6~4.2)	<0.001	2.5 (1.6~4.1)	<0.001
Categorical								
Low-TI	1 (Reference)		1 (Reference)		1 (Reference)		1 (Reference)	
High-TI	4.5 (1.5~13.1)	0.010	4.5 (1.5~13.6)	0.010	10.6 (2.5~44.8)	<0.001	10.8 (2.6~45.5)	<0.001

Model 1: unadjusted. Model 2: adjusted for age, sex, total patency score, distal intimal tear numbers. Abbreviations: TI: tortuosity index; TAE: thoracic aortic enlargement; AAE: abdominal aortic enlargement; HR: hazard ratio; CI: confidence interval.

## Data Availability

The data that support the findings of this study are available from the corresponding author upon reasonable request.

## Supplementary Materials

The following supporting information can be downloaded at: https://www.mdpi.com/article/10.3390/jcm15114139/s1, Figure S1: Bland–Altman plots for inter-observer consistency of morphological measurements; Figure S2. Association between aortic arch TI and aortic enlargement using restricted cubic splines; Table S1: Manual measurement Pprotocol Rreproducibility Aanalysis by Ttwo Oobservers; Table S2: Summary of Mmissing Vvalues and Ppercentages for Aall Aanalyzed Vvariables.

## Abbreviations

TBAD, type B aortic dissection; TEVAR, thoracic endovascular aortic repair; TI, tortuosity index; TAE, thoracic aortic enlargement; AAE, abdominal aortic enlargement; CTA, computed tomography angiography; LSA, left subclavian artery; CLL, centerline length; DTA, descending thoracic aorta; AA, abdominal aorta; TL, true lumen; FL, false lumen; SINE, stent graft-induced new entry; RTAD, retrograde type A dissection; HR, hazard ratio; CI, confidence interval; KM, Kaplan-Meier; RCS, restricted cubic spline; ROC, receiver operating characteristic; AUC, area under the curve; WSS, wall shear stress; CFD, computational fluid dynamics.
