# Supplementary material for: Aortic Arch Tortuosity Index Is Associated with Aortic Enlargement After Thoracic Endovascular Aortic Repair for Left Subclavian Artery Reconstruction Using a Single-Branched Stent-Graft in Type B Aortic Dissection: A Multicenter Retrospective Study
Source: J Clin Med. 2026 May 27;15(11):4139. doi: 10.3390/jcm15114139 (PMC13258805; doi:10.3390/jcm15114139)
Supplement: Supplementary file 1 [file jcm-15-04139-s001.zip › jcm-4291280-supplementary.pdf]

Table S1. Manual measurement protocol reproducibility analysis by two observers.

	Intraclass Correlation Coefficient (ICC)			Bland–Altman		
	Value	95%CI		Mean difference	Limits of Agreement	
		Lower	Upper		Lower	Upper
Diameter-pre-operation-DTA	0.976	0.955	0.987	-0.327	-0.395	3.295
Diameter-post-operation-DTA	0.988	0.977	0.993	-0.321	-3.833	3.191
Diameter-pre-operation-AA	0.970	0.944	0.984	-0.27	-2.108	1.568
Diameter-post-operation-AA	0.986	0.974	0.993	-0.16	-1.658	1.337
Tortuosity Index	0.998	0.997	0.999	0.006	-0.074	0.086

Abbreviations: ICC, intraclass correlation coefficient; CI, confidence interval; DTA, descending thoracic aorta; AA, abdominal aorta.

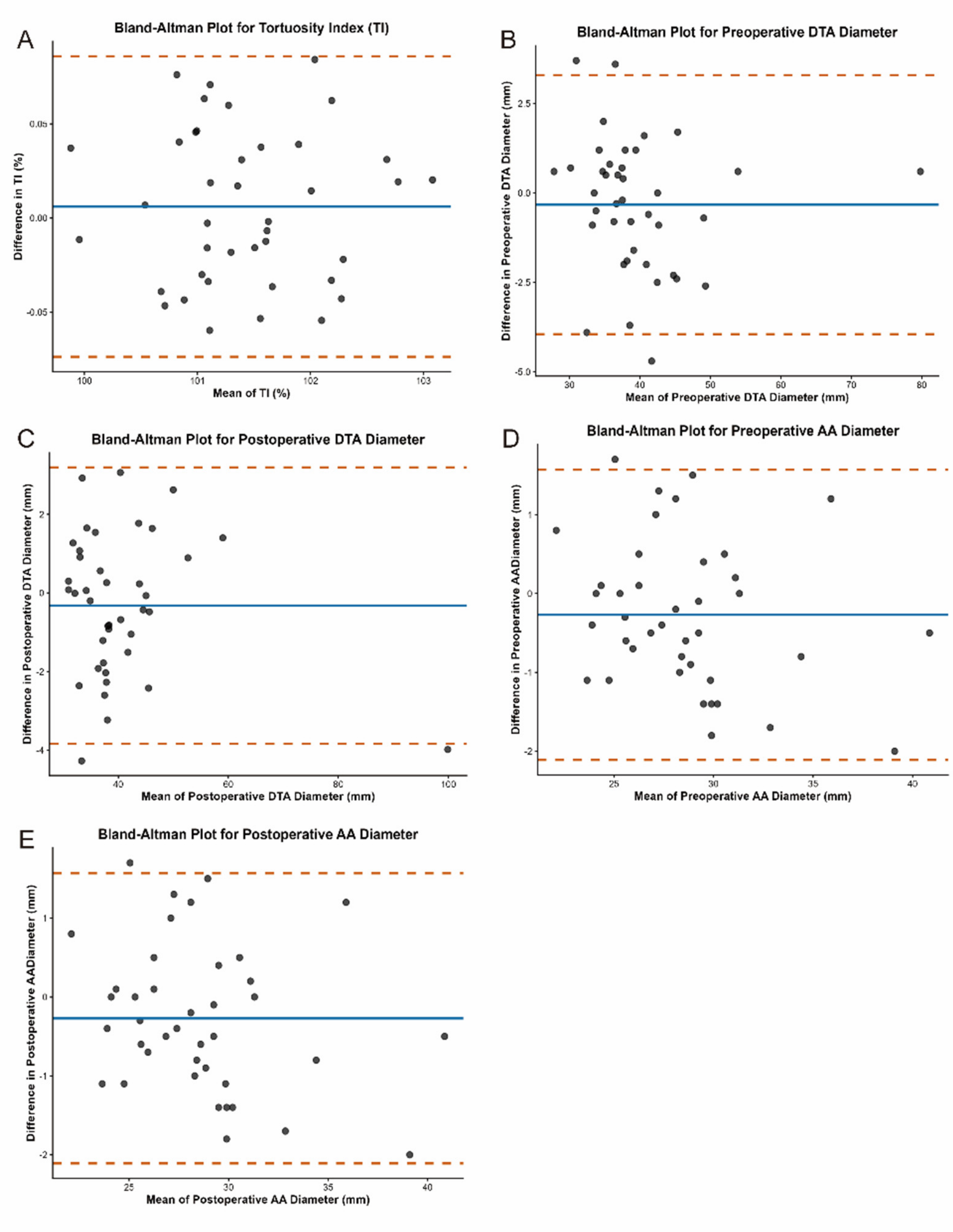

Figure S1. Bland–Altman plots for inter-observer consistency of morphological measurements.

The plots illustrate the agreement between two independent observers for: (A) Tortuosity Index; (B) preoperative DTA diameter; (C) postoperative DTA diameter; (D) preoperative AA diameter; and (E) postoperative AA diameter. The solid blue line represents the mean difference (bias), and the dashed orange lines indicate the 95% limits of agreement (mean ± 1.96 SD). DTA: descending thoracic aorta; AA: abdominal aorta.

Table S2. Summary of missing values and percentages for all analyzed variables.

Variable	Miss. frequence	Miss. percentage%
AAE	0	0
AAE_time	0	0
Age	0	0
TI	0	0
BMI	14	11.6667
Diabetes	0	0
Dissected_length	0	0
Endograft_length	0	0
Gender	0	0
CAD	0	0
Hyperlipemia	0	0
Hypertension	0	0
Phase	0	0
Arch type	0	0
Post_AA_max_FL_diameter	11	9.1667
Post_AA_max_overall_diameter	11	9.1667
Post_AA_max_TL_diameter	11	9.1667
Post_DTA_max_FL_diameter	11	9.1667
Post_DTA_max_overall_diameter	11	9.1667
Post_DTA_max_TL_diameter	11	9.1667
Pre_AA_max_FL_diameter	0	0
Pre_AA_max_overall_diameter	0	0
Pre_AA_max_TLdiameter	0	0
Pre_DTA_max_FL_diameter	0	0
Pre_DTA_max_overall_diameter	0	0
Pre_DTA_max_TL_diameter	0	0
Primary_intimal_tear_diameter	20	16.6667
Smoking	0	0
TAE	0	0
TAE_time	0	0
Death	0	0
Endoleak	0	0
RTAD	0	0
SINE	0	0
Stroke	0	0
RF	0	0
SCI	0	0
Stenosis or occlusion	0	0
Total_Intimal_tear_nubers.	0	0
Distal_intimal_tear_numbers	0	0
Total_thrombosi_score	0	0

Abbreviations: AAE, abdominal aortic enlargement; AA, abdominal aorta; BMI, body mass index; CAD, coronary artery disease; DTA, descending thoracic aorta; FL, false lumen; RF, respiratory failure; RTAD, retrograde type A aortic dissection; SCI, spinal cord ischemia; SINE, stent-graft-induced new entry; TAE, thoracic aortic enlargement; TI, tortuosity index; TL, true lumen.

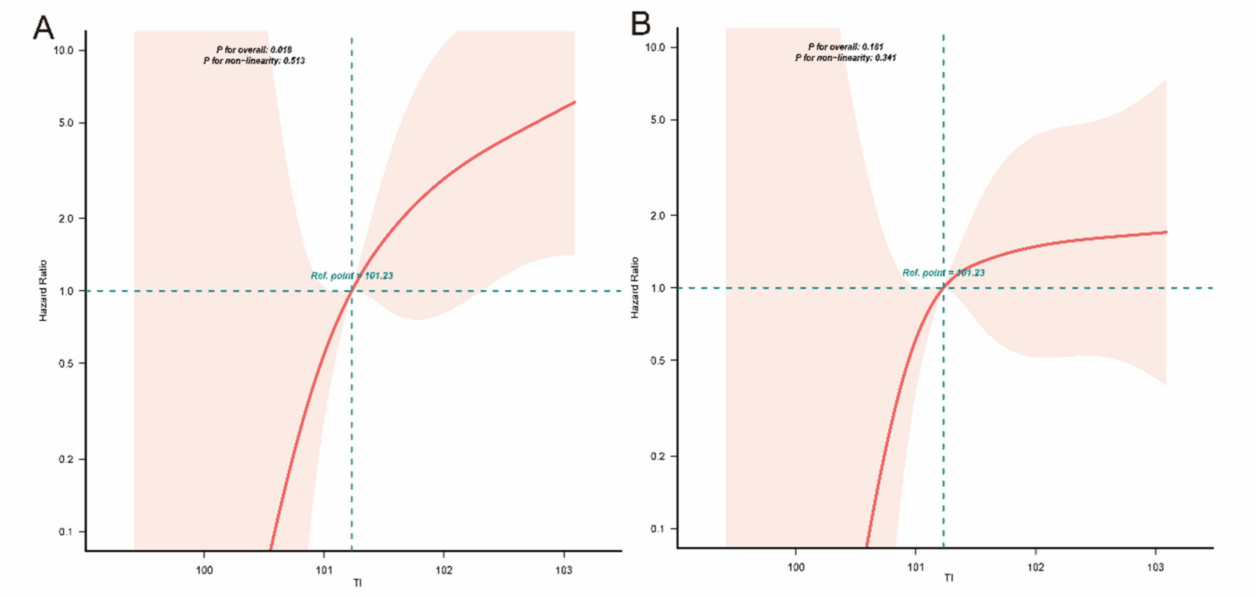

Figure S2. Association between aortic arch TI and aortic enlargement using restricted cubic splines. (A) Thoracic aortic enlargement, (B) abdominal aortic enlargement. TI = tortuosity index.
